# The Impact of Climate-Driven Heat Stress on Bovine Mastitis: A Review of the Po Valley Dairy System

**DOI:** 10.3390/vetsci13070623

**Published:** 2026-06-26

**Authors:** Mario Baratta, Paolo Accornero, Silvia Miretti, Eugenio Martignani

**Affiliations:** 1 Department of Veterinary Science, University of Parma, Via del Taglio 10, 43126 Parma, Italy; 2 Department of Veterinary Sciences, University of Turin, 10124 Torino, Italy; paolo.accornero@unito.it (P.A.); silvia.miretti@unito.it (S.M.); eugenio.martignani@unito.it (E.M.)

**Keywords:** heat stress, mastitis, dairy cattle, temperature–humidity index, Po Valley

## Abstract

This review examines the relationship between climate-driven heat stress (HS) and bovine mastitis in the Po Valley, a key European dairy region characterized by intensive production systems and increasing climatic vulnerability. It aims to contextualize how rising temperature–humidity index (THI) levels influence animal health and productivity. This study synthesizes the current literature on biometeorological conditions, epidemiological trends, and physiological mechanisms linking HS to mastitis. Evidence indicates that prolonged exposure to elevated THI impairs thermoregulation, disrupts endocrine and metabolic balance, and weakens immune function, thereby increasing susceptibility to intramammary infections. Epidemiological data reveal a clear seasonal pattern, with mastitis incidence peaking during summer months and a growing predominance of environmental pathogens. Additionally, HS negatively affects milk yield and quality, amplifying economic losses in dairy systems. The findings highlight that mastitis in this context is not merely an infectious disease but a multifactorial condition shaped by environmental, physiological, and management factors. Overall, this review underscores the need for integrated mitigation strategies, including improved housing, nutrition, genetic selection, and precision monitoring, to enhance resilience. In the face of ongoing climate change, adapting dairy production systems will be essential to safeguard animal welfare, maintain productivity, and ensure the long-term sustainability of the Po Valley dairy sector.

## Simple Summary

Dairy farming in the Po Valley, one of Europe’s most important milk-producing regions, is increasingly affected by hotter and more humid summers due to climate change. These conditions create heat stress in cows, meaning their bodies struggle to cool down, which can weaken their health. This review aims to explain how heat stress contributes to mastitis, a common and costly infection of the udder that reduces milk production and quality, thereby impacting the economic and social sustainability of dairy farms. By analyzing existing research, we found that prolonged exposure to heat and humidity not only lowers cows’ ability to fight infections but also creates conditions that favor the growth of harmful bacteria in their environment. As a result, cases of mastitis tend to rise during the summer months. This study also highlights that modern high-producing cows are particularly vulnerable because of their greater metabolic demands. Understanding this link is important because mastitis leads to economic losses for farmers and can affect animal welfare. The findings suggest that improving farm conditions, nutrition, and animal management can help reduce these risks. Overall, this work provides practical insights to support more resilient and sustainable dairy farming in the face of climate change.

## 1. Introduction

The Po Valley (Pianura Padana) represents the core of the Italian dairy industry and one of the most productive agricultural regions in Europe. This area accounts for a substantial proportion of national milk production, supporting high-value Protected Designation of Origin (PDO) products such as Parmigiano Reggiano and Grana Padano. The intensive nature of dairy farming in this region is characterized by high stocking densities, large herd sizes, and a strong genetic selection for milk yield, primarily in Holstein-Friesian cattle [1,2]. Despite its economic importance, the Po Valley is increasingly recognized as a climate-sensitive system. Its geographical configuration—enclosed between the Alps and the Apennines—creates a basin effect that favors atmospheric stagnation, high humidity, and reduced wind circulation. These conditions contribute to the development of a humid subtropical climate, particularly during summer, which is associated with prolonged periods of elevated temperature–humidity index (THI) [3,4,5].

In recent decades, climate change has intensified these conditions, leading to more frequent and prolonged heatwaves. The increasing occurrence of high THI values, often exceeding the physiological thresholds of dairy cattle, has emerged as a major constraint on productivity, animal welfare, and health [2,6].

Among the various health challenges exacerbated by heat stress (HS), mastitis remains one of the most significant due to its high prevalence and substantial economic impact [7,8].

Mastitis is widely recognized as the most costly disease affecting the dairy industry worldwide. It leads to reduced milk yield, altered milk composition, increased treatment costs, and premature culling [8]. The relationship between HS and mastitis is complex and multifactorial. HS affects not only the external environment but also the internal physiology of the cow, inducing endocrine disruption, oxidative stress, and immunological impairment [1,5]. Furthermore, the modern dairy cow, characterized by high metabolic output, is particularly vulnerable to thermal stress. High-producing animals generate substantial metabolic heat, reducing their capacity to maintain homeothermy [3,6,9].

## 2. Biometeorology of the Po Valley: The Role of the Temperature–Humidity Index (THI)

The Po Valley represents a unique agro-climatic system in Europe, characterized by a combination of high dairy production and challenging environmental conditions. Its geomorphological configuration—enclosed by the Alpine arc to the north and west and the Apennines to the south—creates a semi-closed basin that promotes atmospheric stagnation, high humidity, and limited air circulation. These features contribute to the formation of a microclimate that significantly amplifies HS in dairy cattle, particularly during summer.

In this context, the temperature–humidity index (THI) has emerged as the most widely used biometeorological indicator to quantify thermal stress in livestock. However, in regions such as the Po Valley, the predictive value of THI extends beyond simple thermal load, reflecting the cumulative interaction between temperature, humidity, and exposure duration. This section examines the biometeorological characteristics of the Po Valley, the dynamics of THI under current climatic conditions, and their implications for dairy cow physiology and health.

### 2.1. The Temperature–Humidity Index: Concept and Thresholds

THI integrates ambient temperature and relative humidity into a single parameter to estimate the thermal comfort of animals. It is commonly calculated using the following equation [3]: $THI=\left(1.8\times T+32\right)-\left[\left(0.55-0.0055\times RH\right)\times \left(1.8\times T-26\right)\right]$, where T is ambient temperature (°C) and RH is relative humidity (%). In humid conditions, THI may underestimate heat load; alternative indices such as the Environmental Stress Index (ESI) or heat load index (HLI) have been proposed, though THI remains the most widely used.

Traditionally, a THI threshold of 72 has been considered indicative of the onset of HS in dairy cattle. However, more recent studies have demonstrated that high-producing Holstein cows may experience physiological alterations at significantly lower thresholds, often between 65 and 68 [1,2,3]. This shift reflects the increased metabolic heat production associated with genetic selection for milk yield, as well as the reduced adaptive capacity of modern dairy cows.

HS severity is commonly classified as

Mild: THI 68–72;

Moderate: THI 72–78;

Severe: THI > 78.

Importantly, these thresholds are not absolute and may vary depending on factors such as lactation stage, parity, and acclimatization [4].

### 2.2. Climatic Features of the Po Valley

The Po Valley exhibits a humid subtropical climate (Cfa according to the Köppen classification), characterized by hot summers, high relative humidity, and limited wind circulation. During summer months, the region frequently experiences prolonged periods of high THI, often exceeding critical thresholds for dairy cow thermoneutrality.

Meteorological data from the Lombardia and Emilia-Romagna regions indicate that THI values above 68 can persist for more than 12–16 h per day during July and August, with peaks exceeding 78 during heatwaves [5,6]. Moreover, the occurrence of “tropical nights” (minimum temperature > 20 °C) has increased significantly over the past two decades, limiting nocturnal heat dissipation and preventing physiological recovery [10].

The high humidity typical of the Po Valley further exacerbates thermal stress by reducing the efficiency of evaporative cooling mechanisms. Unlike dry heat conditions, where sweating and panting can effectively dissipate heat, elevated humidity impairs these processes, leading to sustained hyperthermia. These climatic characteristics make the Po Valley particularly prone to chronic HS, as opposed to short-term heat events observed in other regions.

### 2.3. Duration and Accumulation of Heat Load

While peak THI values are important, increasing evidence suggests that the duration of exposure plays an equally critical role in determining the physiological impact of HS. In the Po Valley, prolonged exposure to moderately elevated THI levels can result in cumulative heat load, leading to chronic stress conditions.

Cows exposed to THI values above 68 for extended periods exhibit progressive increases in rectal temperature, respiratory rate, and heart rate, reflecting an inability to dissipate accumulated heat [2]. This condition is often referred to as “chronic heat stress” and is associated with sustained endocrine and metabolic alterations.

Furthermore, the lack of nighttime cooling prevents the restoration of homeostasis, leading to a continuous activation of thermoregulatory mechanisms. This chronic activation has been linked to reduced feed intake, altered energy metabolism, and impaired immune function, all of which contribute to increased disease susceptibility [3,8].

### 2.4. Impact of THI on Dairy Cow Physiology

The physiological response to elevated THI is multifaceted and involves several adaptive mechanisms aimed at maintaining thermal balance. These include increased respiration rate, peripheral vasodilation, reduced feed intake, and increased water consumption.

While these responses are initially adaptive, prolonged activation leads to negative consequences. Reduced feed intake results in decreased energy availability for milk production and immune function, while increased maintenance requirements further exacerbate energy deficits.

HS also induces endocrine changes, including increased cortisol and altered insulin dynamics, which contribute to metabolic inefficiency and immune suppression [1,3]. These physiological alterations form the basis for the mechanisms described in Section 4, linking HS to mastitis susceptibility.

### 2.5. THI and Milk Production

Numerous studies have demonstrated a strong negative correlation between THI and milk yield. In Italian Holstein populations, milk production begins to decline at THI values as low as 68, with losses becoming more pronounced at higher levels [5]. The magnitude of production loss depends on several factors, including duration of heat exposure, genetic potential for milk yield, and availability of cooling systems.

In the Po Valley, milk losses during summer can exceed 10–15%, representing a significant economic burden for dairy farmers [6].

In addition to quantity, milk quality is also affected. HS has been associated with increased somatic cell count (SCC), altered fat and protein content, and reduced cheese-making properties [6].

### 2.6. THI and Disease Risk: Focus on Mastitis

The relationship between THI and mastitis is both direct and indirect. Elevated THI influences disease risk through physiological pathways (immune suppression, oxidative stress, and epithelial disruption) and environmental pathways (bacterial proliferation and increased pathogen load). Epidemiological data from Northern Italy indicate a clear association between high THI periods and increased mastitis incidence, particularly for environmental pathogens such as *Escherichia coli* and *Streptococcus uberis* [11].

### 2.7. Future Perspectives: Climate Change and THI Trends

Climate change projections suggest that the frequency, intensity, and duration of heatwaves in the Po Valley will continue to increase. This trend is expected to exacerbate HS and increase disease incidence. Recent analyses indicate an increase in the number of days exceeding critical THI thresholds [12]. These changes will likely lead to increased chronic HS, production losses, and mastitis incidence. Adapting to these conditions will require technological, nutritional, and genetic strategies.

## 3. Epidemiological Trends of Bovine Mastitis in the Po Valley

The Po Valley represents one of the most intensively farmed dairy regions in Europe, accounting for a substantial proportion of Italian milk production, particularly for Protected Designation of Origin (PDO) cheeses such as Parmigiano Reggiano and Grana Padano. This high-density production system, combined with a unique climatic profile, creates an environment in which mastitis assumes a distinct epidemiological pattern, strongly influenced by seasonal HS dynamics.

Mastitis remains the most prevalent disease affecting adult dairy cattle, with significant economic implications due to decreased milk yield, altered milk composition, increased culling rates, and increased treatment costs. In the Po Valley, epidemiological observations consistently indicate a marked seasonal fluctuation, with incidence peaks occurring during the summer months, in parallel with elevated THI values.

### 3.1. Incidence and Prevalence in Northern Italy Dairy Systems

Large-scale epidemiological studies conducted in Northern Italy provide valuable insights into mastitis dynamics under intensive farming conditions. A multicentric field study across 125 Italian dairy herds reported that clinical mastitis remains highly prevalent, with approximately 30% of herds experiencing monthly incidence rates between 2% and 4%, and over one-third exceeding 4% [13]. These values are consistent with high-producing dairy systems and highlight the persistent burden of mastitis despite advances in herd management and milking hygiene.

More recent analyses based on test-day records from over 800,000 cows in Lombardia confirmed the central role of somatic cell count (SCC) as a proxy for subclinical mastitis and demonstrated a strong negative association between SCC and milk yield. Specifically, a two-fold increase in SCC was associated with an average reduction of approximately 0.83 kg of milk per cow per day, underscoring the substantial production losses linked to udder health impairment [8].

Similarly, a longitudinal cohort study conducted in the Piedmont region, encompassing more than 260,000 cows between 2015 and 2020, identified mastitis as one of the most prevalent health disorders in dairy herds. The risk of mastitis increased significantly with parity (odds ratio > 2.0), stage of lactation, and previous disease occurrence within the same lactation. Importantly, herd-level factors accounted for a substantial proportion of variability, suggesting that environmental and management conditions play a decisive role in disease epidemiology [9].

Taken together, these findings indicate that mastitis in Northern Italy is not solely driven by individual cow susceptibility but emerges from the interaction between animal-level and herd-level determinants, with climate acting as a major modulating factor.

### 3.2. Seasonal Patterns and the Role of HS

**Figure 1:**
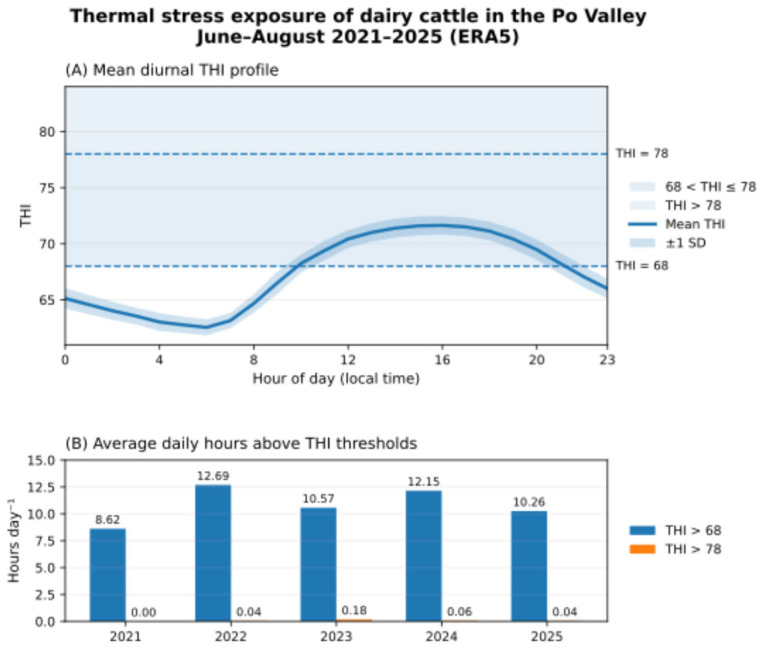
Mean diurnal temperature–humidity index (THI) profile across the Po Valley (Italy) during the warm season (June–August) from 2021 to 2025, derived from ERA5 hourly 2 m air temperature and dew-point temperature data. The solid line represents the multiannual mean THI, while the shaded area indicates interannual variability (±1 SD). Horizontal dashed lines denote the commonly adopted dairy-cattle heat-stress thresholds (THI = 68 and THI = 78). The bar chart summarizes the average daily duration of exposure above each threshold for individual years.

One of the most consistent epidemiological features observed in the Po Valley is the seasonal increase in mastitis incidence during summer. This pattern is closely associated with periods of sustained high THI, often exceeding critical thresholds for dairy cow thermoneutrality [1,2,3]. We processed the climate data available on Copernicus Climate Change Service (C3S); ERA5 hourly data on single levels; and European Centre for Medium-Range Weather Forecasts (ECMWFs), accessed in June 2026 through the Climate Data Store (CDS) (https://cds.climate.copernicus.eu/ accessed on 8 June 2026), over the last 5 years. We show that the mean diurnal THI pattern across the Po Valley exhibited a pronounced daily cycle, with minimum values occurring during the early morning (approximately 62–64 THI units) and maximum values during the afternoon (approximately 71–72 THI units). The threshold associated with the onset of heat stress in dairy cattle (THI = 68) was exceeded for an average of 8.7 to 12.8 h day^−1^ depending on the year. The highest exposure was observed in 2022 (12.83 h day^−1^), followed by 2024 (12.28 h day^−1^). In contrast, exceedance of the severe heat-stress threshold (THI = 78) was rare at the regional scale, averaging less than 0.2 h day^−1^ in all years. These results indicate that moderate heat stress is a recurrent feature of summer conditions in the Po Valley, whereas severe heat-stress conditions are generally limited to short-lived and localized episodes when averaged across the entire region. Figure 1 shows, in a graphic approach, the result of this analysis.

Seasonal analyses of SCC data from Italian Holstein populations have demonstrated a clear “summer peak,” with SCC values increasing progressively from late spring, reaching maximum levels in July and August, and declining during autumn. These trends are not solely attributable to dilution effects from reduced milk yield but reflect a genuine increase in intramammary infections [10].

The climatic conditions of the Po Valley exacerbate this phenomenon. High ambient humidity reduces the efficiency of evaporative cooling, prolonging the duration of HS and limiting nighttime recovery. As a result, cows experience chronic hyperthermia, which has been linked to increased susceptibility to infectious diseases, including mastitis [1,2,3].

Furthermore, HS indirectly influences mastitis epidemiology by modifying animal behavior [11]. Heat-stressed cows tend to spend more time standing and less time lying down, decreasing teat exposure to environmental pathogens present in bedding materials. Simultaneously, reduced feed intake and altered rumination patterns contribute to metabolic stress, further compromising immune function.

However, we need to account for the fact that although the association between elevated THI and mastitis incidence is consistently reported across dairy systems, most available evidence remains observational. Therefore, causality should be interpreted with caution, as several confounding factors may simultaneously influence disease occurrence. Farm hygiene, bedding management, pathogen distribution, parity, genetic background, and individual immune competence can all modulate mastitis risk independently of thermal conditions [12]. Future longitudinal and controlled studies integrating climatic, microbiological, and herd-level variables are needed to clarify the relative contribution of heat stress to mastitis pathogenesis.

### 3.3. Pathogen Distribution and Emerging Trends (2015–2024)

The epidemiology of mastitis in the Po Valley has also evolved in terms of pathogen distribution. Traditionally, contagious pathogens such as *Staphylococcus aureus* and *Streptococcus agalactiae* were dominant; however, recent evidence indicates a shift toward environmental pathogens.

A ten-year surveillance study reported a progressive increase in the prevalence of environmental pathogens, particularly *Streptococcus uberis*, *Escherichia coli*, and *Enterococcus faecium*, between 2015 and 2024. In contrast, the prevalence of classical contagious pathogens has declined, reflecting improvements in milking hygiene and control programs [10].

This epidemiological transition has important implications. Environmental pathogens are strongly influenced by climatic and housing conditions, making their prevalence particularly sensitive to HS. Warm and humid environments promote bacterial proliferation in bedding, manure, and soil, increasing the likelihood of teat contamination.

Moreover, environmental mastitis is often characterized by acute clinical presentations and greater variability in pathogen load, complicating both diagnosis and treatment.

### 3.4. Herd-Level Risk Factors in Intensive Systems

In the Po Valley, dairy farming is characterized by high stocking density, large herd sizes, and high genetic selection for milk yield. These factors contribute to increased metabolic heat production and reduced resilience to environmental stressors.

Even though some studies analyzed the association between the incidence rate of clinical mastitis and herd-level risk factors, such as housing conditions, season, herd size and milk yield, no direct association was found [13]. However, it is worth noting that the study was carried out in Brazil with a different climate, landscape, herd management, and dairy cattle genetic background when compared to the Po Valley. Moreover, the effect of HS should be included in epidemiological studies to assess whether the association could become significant, due to a potential increased risk in dairy cattle subjected to HS.

### 3.5. Economic and Production Impacts

The epidemiological burden of mastitis in the Po Valley is reflected in substantial economic losses. In addition to direct costs related to treatment and discarded milk, indirect losses due to reduced milk yield represent a major component [6,14,15]. Such indirect losses might also include poor reproductive function associated with clinical mastitis [16] but also cumulative effects on lactose content and milk yield in dairy cattle that previously experienced mastitis [17]. Even though clinical mastitis is treated and mammary functional recovery is obtained, there are long-lasting consequences that negatively affect milk production throughout the life of dairy cattle. Moreover, mastitis increases the risk of premature culling [18], especially in high-producing cows, leading to additional replacement costs.

### 3.6. Future Epidemiological Perspectives Under Climate Change

According to the annual report “Climate in Italy in 2023” [19], significant trends of temperature increases (+0.45 ± 0.05 °C over 10 years) in northern Italy, where the Po Valley is located, and a general trend of summer temperature increase (+0.60 ± 0.09 °C over 10 years) are evident. Therefore, climate projections indicate that the Po Valley will experience more frequent and prolonged heatwaves in the coming decades. This trend is likely to exacerbate existing challenges in dairy production and further increase the incidence of mastitis. It has already reported the effect of seasonal HS conditions on both bulk milk traits and traits of test-day records collected in parallel from farms located on the plain and in mountain areas in Italy [6], with even mild HS conditions having detrimental effects on the quality of milk, while the effect on yield was negligible. The effect of THI on milk quality was more consistent although less severe in bulk milk than in test-day records. Future epidemiological research should focus on integrating climatic data with herd health records and developing predictive models for disease risk.

## 4. Physiological Mechanisms of Action

HS represents a pervasive and multifactorial challenge for dairy cattle, particularly in intensive production systems such as those characterizing the Po Valley. In this region, prolonged exposure to elevated temperature–humidity index (THI) values, often combined with insufficient nocturnal cooling, results in a chronic state of thermal overload. Unlike acute stressors, HS induces a sustained disruption of homeostasis that extends beyond thermoregulation, profoundly affecting endocrine signaling, cellular redox balance, intestinal integrity, and immune competence. Within this complex physiological framework, mastitis emerges not simply as an infectious disease but also as the clinical manifestation of systemic dysregulation [1,2].

The overarching systemic response is coordinated by a central regulatory hub connecting multiple systems (brain, HPA axis, and gut). The link between HS and mastitis is increasingly recognized as a consequence of interconnected biological pathways rather than isolated mechanisms. Endocrine alterations, oxidative damage, and immune dysfunction act synergistically, while recent advances [20] highlight the pivotal role of the gut–mammary axis in mediating systemic inflammation. These processes collectively compromise both the structural defenses of the mammary gland and the functional efficiency of the immune system, ultimately increasing the incidence and severity of intramammary infections (IMIs). After systemic cortisol release and subsequent intestinal integrity disruption, LPSs translocate into blood circulation and activate mammary macrophages via Toll-like receptor 4 (TLR4), triggering a pro-inflammatory cytokine storm (IL-1β, TNF-α, and IL-6), leading to glandular inflammation and dysfunction. At the same time, ROS accumulation due to stress experience activates the cascade, resulting in direct mammary epithelial damage and mitochondrial dysfunction (liver–mitochondria–efficiency feedback loop). It also converges with the LPS translocation/leaky gut path. Both pathways converge, presenting the multifactorial nature of HS-induced mastitis, culminating in distinct glandular inflammation and dysfunction, mammary epithelial damage, and the final state of mastitis.

**Figure 2:**
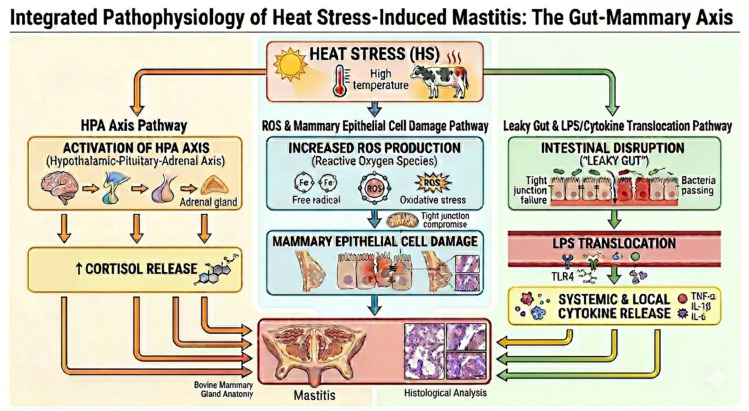
Comprehensive integrated pathophysiology of heat stress (HS)-induced mastitis. This diagram illustrates a complex multi-organ model detailing parallel, converging physiological pathways driven by heat stress (HS) to induce localized mastitis in dairy cattle. (**Left**) (HPA Axis Pathway): Follows the sequence from HPA axis activation and systemic cortisol release, through localized intestinal integrity disruption (Leaky Gut). (**Right**) (ROS and Oxidative Stress Pathway): The induction of reactive oxygen species (ROS) and oxidative stress. (**Central**) Histological Assessment: Histological analysis of normal mammary tissue versus inflamed tissue with leukocyte infiltration and dysfunction.

Figure 2 summarizes the various mechanisms of action involved.

### 4.1. Endocrine Disruptions: Cortisol and Prolactin

One of the earliest physiological responses to HS is the activation of the hypothalamic–pituitary–adrenal (HPA) axis. Exposure to high environmental temperatures stimulates hypothalamic release of corticotropin-releasing hormone, leading to increased secretion of adrenocorticotropic hormone and, consequently, cortisol [20]. While acute increases in cortisol are essential for maintaining metabolic stability, chronic hypercortisolemia—commonly observed under prolonged HS conditions—exerts detrimental effects on immune regulation and tissue homeostasis [21].

Cortisol is a potent immunomodulator, and its sustained elevation leads to a progressive impairment of both innate and adaptive immune responses. Experimental and field studies have demonstrated that heat-stressed cows exhibit reduced lymphocyte proliferation; altered leukocyte trafficking; and suppression of key inflammatory signaling pathways, including NF-kB [22]. These alterations result in a diminished capacity to mount an effective immune response against invading pathogens.

In the context of mastitis, cortisol-mediated effects are particularly relevant during the early stages of infection. The recruitment of neutrophils to the mammary gland is a critical determinant of infection outcome; however, HS has been shown to impair leukocyte adhesion and transmigration across endothelial barriers, delaying immune intervention [23]. This delay creates a temporal window during which pathogens can colonize the teat canal and establish infection.

**Figure 3:**
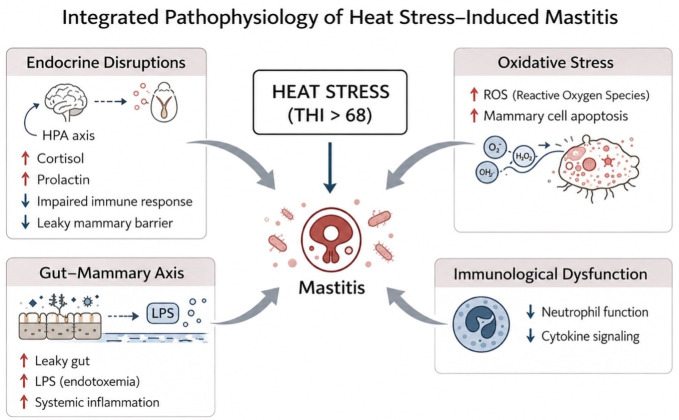
Heat stress (temperature–humidity index, THI > 68) triggers endocrine disruptions (cortisol and prolactin), oxidative stress, gut–mammary axis dysfunction, and immunological impairment. These interconnected pathways reduce immune competence, increase systemic inflammation, and compromise mammary barrier integrity, ultimately promoting the onset and severity of mastitis.

In parallel with cortisol, prolactin plays a crucial role in maintaining mammary gland function and epithelial integrity. HS disrupts prolactin secretion and receptor sensitivity, leading to a condition of functional dysregulation despite elevated circulating hormone levels [21]. This phenomenon is associated with impaired tight junction maintenance within mammary epithelial cells, resulting in increased permeability of the blood–milk barrier. Figure 3 summarizes the conceptual model of this mechanism of action.

The integrity of this barrier is essential for preventing the translocation of pathogens and inflammatory mediators. Its disruption facilitates the movement of ions and proteins between blood and milk, altering milk composition and creating a microenvironment more conducive to bacterial growth. Moreover, prolactin dysregulation may impair epithelial cell renewal and repair, further weakening mammary defenses [24].

These endocrine changes are further compounded by metabolic alterations induced by HS. Reduced dry matter intake leads to a state resembling negative energy balance, even during mid-lactation, with downstream effects on insulin and insulin-like growth factor-1 (IGF-1) signaling [25]. These metabolic–endocrine interactions contribute to immunosuppression and reduced resilience of mammary tissue, reinforcing susceptibility to mastitis.

### 4.2. Oxidative Stress and Mammary Cell Apoptosis

A central feature of HS physiology is the induction of oxidative stress, resulting from an imbalance between the production of reactive oxygen species (ROS) and the capacity of antioxidant defense systems. Elevated temperatures accelerate mitochondrial activity and disrupt electron transport chains, leading to increased generation of ROS such as superoxide anion, hydrogen peroxide, and hydroxyl radicals [26].

Simultaneously, HS has been associated with a reduction in antioxidant enzyme activity, including superoxide dismutase, glutathione peroxidase, and catalase [26]. The resulting oxidative imbalance has profound implications for cellular integrity, particularly in the mammary gland, which is characterized by high metabolic activity and continuous biosynthetic demand.

Oxidative damage to mammary epithelial cells (MECs) manifests through lipid peroxidation, protein oxidation, and DNA damage. These processes compromise membrane integrity, alter cellular signaling pathways, and impair the functional capacity of the epithelium [27]. Of particular importance is the disruption of tight junction proteins, such as claudins and occludins, which play a key role in maintaining the blood–milk barrier.

The breakdown of tight junctions leads to increased paracellular permeability, allowing the leakage of lactose into the bloodstream and the influx of sodium and chloride into milk. These changes are reflected in increased milk electrical conductivity and are commonly associated with mastitis [28].

Beyond structural damage, oxidative stress activates apoptotic pathways within MECs. The intrinsic (mitochondrial) pathway is triggered by ROS-induced mitochondrial dysfunction, leading to the release of cytochrome c and activation of caspases [29]. Excessive apoptosis reduces the population of functional epithelial cells, impairing milk production and weakening the physical barrier against pathogens.

Furthermore, apoptotic cell debris may act as a substrate for bacterial proliferation and contribute to local inflammatory responses, thereby exacerbating the risk and severity of mastitis.

It has to be taken into account however that during bovine mastitis, the fate of mammary epithelial cells is primarily determined by two distinct pathways of cell death: apoptosis and necrosis [30]. Distinctly differentiating these mechanisms is crucial, as their morphological and immunological outcomes impact the severity and resolution of mammary inflammation differently. While apoptosis allows for the silent clearance of damaged MECs without disrupting the blood–milk barrier, necrosis acts as a potent pro-inflammatory driver within the mammary gland. When MECs undergo necrosis, the loss of membrane integrity results in the uncontrolled release of intracellular contents into the extracellular space. These contents include Damage-Associated Molecular Patterns (DAMPs)—such as high-mobility group box 1 (HMGB1) protein, heat shock proteins, and purine metabolites (ATP). Consequently, extensive necrosis not only perpetuates a vicious cycle of neutrophil recruitment and oxidative stress but also leads to the enzymatic destruction of the extracellular matrix. This exacerbates mammary tissue damage, delays involution or healing, and can ultimately lead to permanent loss of milk-producing alveolar structures.

### 4.3. The Gut–Mammary Axis: Leaky Gut and Endotoxemia

In recent years, the role of the gastrointestinal tract in mediating systemic responses to HS has gained increasing attention. The concept of the gut–mammary axis provides a framework for understanding how intestinal dysfunction can influence mammary health.

During HS, blood flow is preferentially redirected toward peripheral tissues to facilitate heat dissipation. This redistribution results in reduced perfusion of the gastrointestinal tract, leading to hypoxia and metabolic stress in enterocytes [31,32]. As a consequence, the integrity of the intestinal barrier is compromised, resulting in increased permeability—a condition commonly referred to as “leaky gut” [33].

The loss of barrier function allows the translocation of microbial components, particularly lipopolysaccharides (LPSs), into systemic circulation [34]. LPSs are potent endotoxins that activate Toll-like receptor 4 (TLR4), triggering intracellular signaling pathways that culminate in the production of pro-inflammatory cytokines such as TNF-alpha, IL-1beta, and IL-6 [34].

This state of endotoxemia induces a chronic low-grade inflammatory response that persists under prolonged HS conditions. Importantly, this systemic inflammation has significant implications for mammary gland physiology. Circulating cytokines increase vascular permeability and promote the recruitment of immune cells to mammary tissue, but in a dysregulated manner.

As a result, the mammary gland becomes “primed” for inflammation, exhibiting exaggerated responses even to minor bacterial challenges. This hypersensitivity can lead to increased tissue damage and higher somatic cell counts, even in cases of low pathogen load [35].

The gut–mammary axis therefore represents a critical link between environmental stress and mammary disease, particularly in conditions such as those observed in the Po Valley, where HS is prolonged and recurrent.

### 4.4. Immunological Compromise: Neutrophil Function and Cytokine Signaling

The immune system is the final line of defense against mastitis, and its impairment under HS conditions plays a decisive role in disease susceptibility. Among immune cells, neutrophils are particularly important due to their rapid response and potent antimicrobial activity.

**Figure 4:**
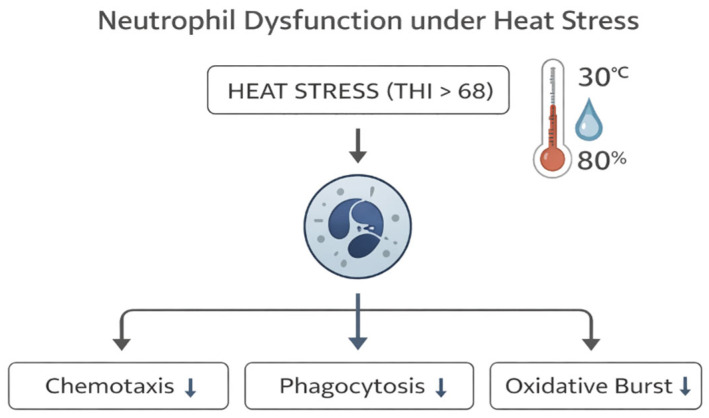
Effects of heat stress on neutrophil function in dairy cows. Exposure to elevated temperature–humidity index (THI > 68) leads to neutrophil dysfunction, characterized by reduced chemotaxis, impaired phagocytosis, and decreased oxidative burst activity. These alterations compromise innate immune defense and increase susceptibility to intramammary infections.

HS has been shown to impair neutrophil function at multiple levels; see Figure 4. One of the most consistently reported effects is a reduction in chemotaxis, the process by which neutrophils migrate toward sites of infection [22]. This impairment is associated with altered expression of chemokine receptors and cytoskeletal dysfunction, resulting in delayed immune responses.

In addition to impaired migration, neutrophils from heat-stressed cows exhibit reduced phagocytic capacity, limiting their ability to engulf and eliminate pathogens [26]. This defect is further compounded by a reduction in oxidative burst activity, a key bactericidal mechanism involving the production of reactive oxygen intermediates.

The combined effect of these alterations is a significant reduction in the efficiency of pathogen clearance, particularly against environmental bacteria such as *Escherichia coli* and *Streptococcus uberis*, which are prevalent in summer conditions.

HS also disrupts cytokine signaling, leading to an imbalance between pro-inflammatory and anti-inflammatory responses. While basal levels of cytokines such as TNF-alpha and IL-6 are elevated, the ability to mount an effective acute response to infection is impaired [34]. This paradoxical condition results in chronic low-grade inflammation coupled with inadequate pathogen defense.

The dysregulation of cytokine networks further affects the coordination between innate and adaptive immunity, reducing the overall resilience of the immune system and contributing to increased mastitis incidence.

### 4.5. Integrated Pathophysiological Model

The mechanisms described above do not operate in isolation but form an interconnected network of physiological disturbances. Endocrine dysregulation alters immune signaling and metabolic priorities; oxidative stress damages epithelial barriers; intestinal permeability induces systemic inflammation; and immune dysfunction prevents effective pathogen clearance.

In the Po Valley, where HS exposure is prolonged and often uninterrupted, these processes become chronic, transforming adaptive responses into pathological conditions. The resulting scenario is characterized by a structurally compromised mammary gland, a dysregulated immune system, and a pro-inflammatory systemic environment.

This integrated model provides a comprehensive explanation for the marked seasonal increase in mastitis observed in dairy herds during summer months. It also underscores the importance of considering HS not merely as an environmental stressor but as a central driver of pathogenesis. However, it is worth noting that such a model relies on a set of effects that in the literature are examined as individual factors. Moreover, some of the mechanisms that we described are demonstrated in contexts that are not exclusive to HS, such as LPS translocation in the blood stream due to the leaky gut condition. As plausible and interesting this complex model appears, there is a lack of multifactorial analyses that would examine, in a controlled experimental setup, all of the axis that we described when cattle is exposed to high THI.

## 5. Mitigation Strategies: Reducing HS and Mastitis Risk

The increasing impact of HS on dairy production systems in the Po Valley necessitates the implementation of effective mitigation strategies. Given the multifactorial nature of HS, successful interventions must address environmental, nutritional, and genetic components simultaneously. Moreover, because mastitis risk is closely linked to both host physiology and environmental pathogen pressure, mitigation strategies should aim not only to reduce thermal load but also to preserve immune competence and udder health.

### 5.1. Environmental and Housing Interventions

Environmental management represents the first and most immediate approach to mitigating HS. The primary objective is to enhance heat dissipation and reduce the thermal load experienced by dairy cows [36].

#### 5.1.1. Ventilation and Cooling Systems

Mechanical ventilation systems, including fans and tunnel ventilation, are widely used to improve air circulation in dairy barns. When combined with evaporative cooling systems such as sprinklers or mist sprayers, these technologies can significantly reduce body temperature and improve animal comfort [36].

Studies have shown that cows exposed to combined fan and sprinkler systems exhibit lower rectal temperatures, reduced respiration rates, and increased milk yield compared to non-cooled animals [37]. Importantly, these systems also contribute to improved immune function, indirectly reducing susceptibility to mastitis.

#### 5.1.2. Bedding and Hygiene Management

HS promotes bacterial proliferation in bedding materials, increasing environmental pathogen load. Therefore, maintaining dry and clean bedding is essential during summer months. Frequent replacement of bedding materials, proper drainage, and the use of inorganic bedding (e.g., sand) have been associated with lower mastitis incidence [38]. Additionally, optimizing stocking density and improving stall design can reduce cow-to-cow contact and minimize teat contamination [39].

### 5.2. Nutritional Strategies

Nutritional interventions play a critical role in mitigating the effects of HS by supporting metabolic function and immune competence.

#### 5.2.1. Energy Density and Feed Intake

Heat-stressed cows typically reduce dry matter intake (DMI), leading to negative energy balance. Increasing the energy density of the diet through the inclusion of highly digestible feeds and rumen-protected fats can help compensate for reduced intake [40].

#### 5.2.2. Antioxidants and Immune Support

Given the role of oxidative stress in HS, supplementation with antioxidants such as vitamin E, selenium, and polyphenols has been shown to improve immune function and reduce oxidative damage [41]. Recent studies also suggest that specific feed additives, including yeast cultures and probiotics, may enhance gut integrity and reduce endotoxemia, thereby supporting the gut–mammary axis [42].

### 5.3. Genetic and Breeding Approaches

Long-term mitigation of HS requires the selection of animals with improved heat tolerance. Genetic approaches focus on identifying traits associated with thermoregulation, such as lower body temperature, improved feed efficiency under HS, and resilience in milk production [43]. Genomic selection programs have begun to incorporate heat tolerance indices, allowing breeders to select animals that maintain productivity under elevated THI conditions [44]. Crossbreeding strategies may also offer advantages by combining high productivity with improved adaptability. However, we have to admit that heat tolerance often negatively correlates with milk yield; thus, genomic selection is promising but not a standalone solution [45].

### 5.4. Precision Livestock Farming and Monitoring

Advances in precision livestock farming (PLF) technologies provide new opportunities for early detection and management of HS. Sensors capable of monitoring body temperature, activity, and rumination can be used to identify HS in real time [46]. Similarly, automated milking systems and milk sensors allow continuous monitoring of SCC and milk conductivity, facilitating early detection of mastitis. Integrating these data with climatic information (e.g., THI) enables the development of predictive models for disease risk.

### 5.5. Integrated Management Approach

Given the complexity of HS, no single intervention is sufficient to fully mitigate its effects. Instead, an integrated approach combining environmental, nutritional, genetic strategies and clinical observations such as clinical indicators such as mastitis and lameness is required [47]. In the Po Valley, where climatic conditions are particularly challenging, the effectiveness of mitigation strategies depends on their ability to address both thermal stress and pathogen exposure simultaneously. For example, improving ventilation without addressing bedding hygiene may reduce heat load but fail to control mastitis risk.

Therefore, a successful management should aim to

- Reduce THI exposure;
- Maintain immune competence;
- Limit environmental pathogen load.

### 5.6. Future Perspectives

As climate change continues to intensify HS conditions, the importance of mitigation strategies will increase. Future research should focus on

- Developing climate-resilient dairy systems;
- Improving predictive models for HS and mastitis;
- Evaluating the long-term effectiveness of mitigation strategies.

In addition, policy-level interventions may be required to support farmers in adopting advanced cooling technologies and sustainable management practices. Despite their demonstrated effectiveness, many mitigation strategies may face important barriers to adoption. Advanced cooling systems, sensor-based monitoring platforms, and genomic selection programs often require substantial capital investment, technical expertise, and infrastructural adaptation. These requirements may limit implementation, particularly in small and medium-sized dairy farms. Consequently, future adaptation frameworks should consider not only biological effectiveness but also economic feasibility, scalability, and farm-specific constraints. Greater attention should be devoted to cost-effective interventions that can be progressively integrated into existing management systems.

## 6. Conclusions

HS represents a major and growing challenge for dairy production systems in the Po Valley, with significant implications for animal health, welfare, and productivity. Among the various health issues associated with HS, mastitis emerges as a climate-sensitive disease whose incidence is strongly influenced by environmental conditions and host physiological responses.

This review highlights how elevated THI values, particularly when sustained over time, contribute to increased mastitis risk through a combination of environmental and physiological mechanisms. On the one hand, high temperature and humidity favor bacterial proliferation and increase pathogen exposure. HS induces endocrine disruption, oxidative stress, intestinal barrier dysfunction, and immune suppression, all of which compromise the ability of the mammary gland to resist infection.

The epidemiological patterns observed in the Po Valley, including the marked seasonal increase in mastitis during summer, can therefore be understood as the result of a complex interaction between climate, management, and host biology.

Mitigation strategies must adopt an integrated approach, combining environmental cooling, nutritional support, genetic selection, and precision monitoring technologies. Such strategies are essential not only to reduce thermal stress but also to maintain immune competence and limit pathogen pressure.

In the context of ongoing climate change, the development of resilient dairy systems will require continuous adaptation and innovation. Future research should focus on integrating climatic data with herd health monitoring and on developing predictive models to support decision-making at the farm level.

Several important knowledge gaps remain. First, the long-term physiological and immunological consequences of repeated heat-stress exposure across multiple lactations are still poorly understood. Second, limited information is available regarding potential trade-offs between genetic selection for heat tolerance and production performance. Third, the emerging role of the gut–mammary axis requires further validation under commercial farming conditions. Finally, relatively few studies have evaluated the economic efficiency of mitigation strategies through comprehensive cost–benefit analyses. Addressing these gaps will be essential for designing sustainable adaptation strategies under future climate scenarios. Ultimately, improving our understanding of the relationship between HS and mastitis will be crucial for ensuring the sustainability and competitiveness of dairy production in the Po Valley and other climate-sensitive regions.

## Data Availability

No new data were created or analyzed in this study. Data sharing is not applicable to this article.
